# Dynamic Augmented Reality Cues for Telementoring in Laparoscopic Surgery: Usability Study

**DOI:** 10.2196/86566

**Published:** 2026-03-04

**Authors:** Jhasketan Padhan, Hawa Hamza, Dehlela Shabir, Julien Abinahed, Panagiotis Tsiamyrtzis, Zhigang Deng, Nikhil V Navkar

**Affiliations:** 1 Itqan Clinical Simulation and Innovation Center, Hamad Medical Corporation, PO Box 3050, Doha, Qatar; 2 Artificial Intelligence Research and Innovation Hub, Hamad Medical Corporation, PO Box 3050, Doha, 3050, Qatar, 974 77606674; 3 Department of Mechanical Engineering, Politecnico di Milano, Milan, Italy; 4 Department of Statistics, Athens University of Economics and Business, Athens, Greece; 5 Department of Computer Science, University of Houston, Houston, TX, United States

**Keywords:** laparoscopy, minimally invasive surgical procedure, telementoring, augmented reality, dynamic visual cues

## Abstract

**Background:**

Surgical telementoring enables a remote expert surgeon (mentor) to guide an operating surgeon (mentee) during surgery and facilitates the transfer of surgical skills. However, commonly used audio and static visual cues are inadequate to demonstrate complex tool-tissue interactions. To overcome this limitation, dynamic augmented reality (AR)–based visual cues are overlayed on the operative field to demonstrate precise instrument movements.

**Objective:**

The objective of this work was to evaluate dynamic AR cues and identify the most suitable cue that effectively demonstrates the required motions of surgical instruments during laparoscopy.

**Methods:**

A user study was conducted in a simulated environment among mentor-mentee pairs using 3 dynamic AR cues (hand gestures, a 3D pointer, and a virtual tool). The task assessed how closely the mentee was able to follow the mentor. The outcomes measured were (1) dynamic time warping distance, representing the closeness of the paths followed; (2) angular error in tooltip orientation; and (3) the NASA Task Load Index, assessing cognitive workload during telementoring.

**Results:**

Telementoring using the virtual tool resulted in a reduced dynamic time warping distance compared with hand gestures (*P*<.01 for 12/13, 92.3% of the trials) and the 3D pointer (*P*<.05 for 11/13, 84.6% of the trials). Lower orientation error was also noted while using the virtual tool as compared with hand gestures (*P*<.05 for 5 of 6 poses). There were no substantial differences in the NASA Task Load Index scores.

**Conclusions:**

Use of a virtual tool (as a dynamic AR cue) enabled the mentee to follow the mentor’s instructions with fewer errors than both hand gestures and a 3D pointer without increasing cognitive workload. Further research is needed to assess the clinical effectiveness of the virtual tool during live surgery.

## Introduction

A growing need for expert surgeons’ guidance during laparoscopic surgery necessitates the adoption of telementoring technologies [1]. During telementoring, an expert surgeon (mentor) can guide an operating surgeon (mentee) in real time even if they are continents apart. Operating room footage is sent to the remote mentor, who then provides instructions to the mentee using audio cues. Further enhancing the mode of instruction, the telementoring system may also integrate augmented reality (AR) technologies, whereby computer-simulated virtual objects are superimposed on the user’s field of view [2]. This includes the use of static AR-based visual cues such as annotations or *telestrations* (ie, sketches or illustrations drawn over a video feed by the mentor in real time) overlaid on the mentee’s operative view displayed on a screen. This is useful during in-person guidance as well, as the mentor can point at anatomical structures instead of describing them verbally [3]. However, static AR cues remain stationary within the operating field, and the mentor may not be able to communicate the required tool positions, orientations, and movements. To overcome this limitation, the use of dynamic AR cues, including but not limited to the mentor’s hand gestures, a 3D pointer, and a virtual tool, has been proposed [4]. The remotely located expert surgeon can communicate with the mentee by controlling these cues in real time, thereby demonstrating complex tool-tissue interactions required during laparoscopic surgery. This can also be used to enhance in-person guidance, thereby eliminating the need for the mentor surgeon to take control of the instruments to demonstrate specific tool movements.

Advancements in dynamic AR cues for telementoring during minimally invasive surgery (laparoscopic and robot assisted) have been tested extensively (Table 1). They include procedures such as cholecystectomy, prostatectomy, and suturing in various clinical settings such as virtual simulator, synthetic phantom, animals (cadavers), and humans (live). The dynamic AR cues frequently used for guiding mentees during minimally invasive surgeries can be categorized as hand gestures, pointers, or surgical tools [5]. While some of the studies have provided a description of the technologies used, others have compared dynamic AR cues with different modes of mentoring. They include assessment of hand gestures with audio cues [3,6,7] or in-person guidance [8], 3D pointers with audio cues [9,10], and virtual tools with in-person guidance [11-15]. A comparison of dynamic AR cues against static 2D telestrations has also been conducted [16]. As shown in Table 2, previous articles have already reported favorable results while using dynamic AR cues when compared with standard practices such as audio cues, in-person guidance, and static 2D telestrations. Both hand gestures and 3D pointers have shown improved performance compared with audio cues. Virtual tools have resulted in comparable performance to and lower operating time than that with in-person guidance. In these studies, all 3 dynamic AR cues were preferred over static 2D telestrations. While some of the related studies have introduced these telementoring technologies in cadaveric and live surgical settings, they have not objectively compared the 3 dynamic AR cues, namely, hand gestures, 3D pointers, and virtual tools, against each other. To the best of our knowledge, there are no existing studies that have performed a comparative analysis of the different dynamic AR cues. To enhance telementoring and improve the learning experience for the mentee, there is a need to standardize and optimize the use of dynamic AR cues. This requires comparing different types of dynamic AR cues among themselves to identify the most effective modality for the mentor to render the information to the mentee during surgical telementoring.

**Table 1.** Related works on dynamic augmented reality (AR) cues during minimally invasive surgical telementoring*.*

Telementoring system	Dynamic AR cue			Comparison	Clinical setting	Outcome
	Hand gestures	3D pointer	Virtual tool			
iSurgeon [3,6,7,17]	✓			Audio cue	Cholecystectomy on animals (cadavers)	Better GOALS^a^ and OSATS^b^ scores, fewer complications (*P*<.001), and reduced workload (NASA-TLX^c^; *P*<.02)
HoloPointer [9,10]		✓		Audio cue	Cholecystectomy on virtual simulator and humans (live)	Improved movement economy (*P*=.047), error rate (*P*=.047), and performance (*P*=.03) and favorable ratings by participants
Long et al [18]	✓		✓	—^d^	Prostatectomy on humans (live)	Successful execution of telementoring system
Shabir et al [11-13,19,20]			✓	In-person guidance	Suturing on synthetic phantom	Comparable performance among groups receiving telementoring and in-person guidance
Lowry et al [14]			✓	No cue; in-person guidance	Suturing and basic tasks on synthetic phantom	Comparable performance among groups receiving telementoring and in-person guidance
Proximie [8,21]	✓			In-person guidance	Prostatectomy and aquablation on humans (live)	Overall favorable ratings by participants
Feng et al [22,23]		✓		Audio cue	Cholecystectomy on synthetic phantom	Better movement economy (*P*=.01) and performance (*P*<.001)
Jarc et al [16,24]	✓	✓	✓	Static visual cue (2D telestration)	Suturing and basic tasks on animals (live)	3D tools were preferred over static visual cues by the participants
VIPAR^e^ platform [25]	✓	✓		—	Ventriculostomy on humans (live)	Successful execution of VIPAR platform
ART^f^ [15]			✓	In-person guidance	Suturing on synthetic phantom	Shorter operating time (*P*=.01) and fewer failed attempts while using ART

a GOALS: Global Operative Assessment of Laparoscopic Skills.

b OSATS: Objective Structured Assessment of Technical Skills.

c NASA-TLX: NASA Task Load Index.

d Not applicable.

e VIPAR: virtual interactive presence and AR.

f ART: AR telementoring.

**Table 2.** Advantages of dynamic augmented reality (AR) cues over conventional mentoring methods.

Dynamic AR cue	Comparison		
	Audio cue	In-person guidance	Static 2D telestration
Hand gestures	Better performance score, fewer complications, and reduced workload [3,6,7,17]	Favorable ratings by participants [8,21]	Preferred over static visual cues by the participants [16,24]
3D pointer	Improved movement economy, performance [9,10,22,23], and error rate and favorable ratings by participants [9,10]	—^a^	Preferred over static visual cues by the participants [16,24]
Virtual tool	—	Similar performance [11-14,19,20], shorter operating time, and fewer failed attempts [15]	Preferred over static visual cues by the participants [16,24]

a Not available.

In this study, we aimed to quantitatively assess the dynamic AR cues during telementoring and compare the mentee’s performance while receiving instructions in each mode, namely, hand gestures, a 3D pointer, or a virtual tool. In the following sections, we present the experimental setting used for this study, the results obtained during the trial, and a discussion of the major implications of using dynamic AR cues for telementoring during laparoscopic surgery.

## Methods

### Participant Enrollment

A user study was conducted among mentor-mentee pairs as participants to evaluate the dynamic AR cues for telementoring. Participants were recruited from the Department of Surgery at Hamad General Hospital in Qatar. They were researchers working on the development of surgical technologies. None of them had prior experience performing real surgeries; however, they had experience using surgical simulators and were familiar with the hand-eye coordination required for maneuvering laparoscopic tools. Hand-eye coordination is crucial for laparoscopic tasks, especially when the operative field is displayed to the user on a 2D screen [26]. A total of 10 participants as mentees were included in the user study. One participant, a senior researcher from the Department of Surgery who was familiar with the telementoring system, was recruited as the mentor to demonstrate the required laparoscopic instrument motions to the mentees. The mentor was well versed in using user interfaces at the mentor’s site to demonstrate the required motion of a laparoscopic tool using different dynamic AR cues.

### Setup at the Mentor and Mentee Sites

**Figure 1.**
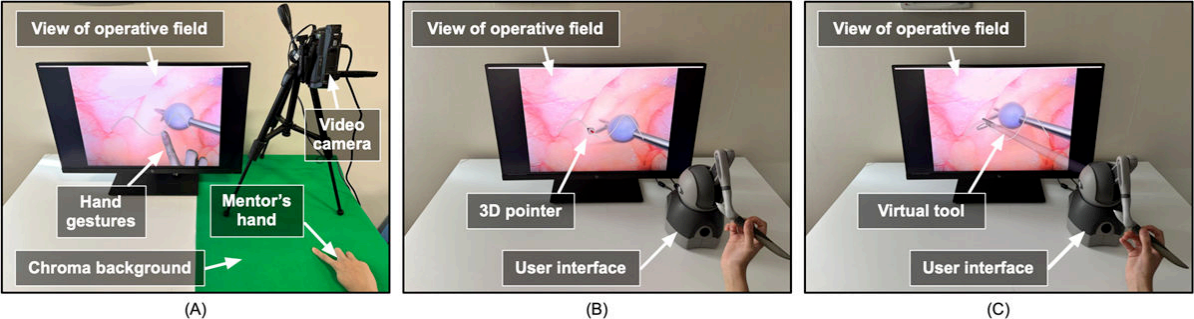
The setup at the mentor’s site to generate the 3 types of dynamic augmented reality cues: (A) hand gestures, (B) a 3D pointer, and (C) a virtual tool.

The mentor used 3 different dynamic AR cues, namely, *hand gestures*, a *3D pointer*, and a *virtual tool*, to guide the mentees. The setup at the mentor’s site for generating each of the dynamic AR cues is shown in Figure 1. To capture hand gestures (Figure 1A), the mentor moved the hand over a chroma background, and a camera was used to acquire the movements. The background was then removed from the acquired video, and the hand gestures were overlaid onto the live view of the operative field. In the case of the 3D pointer (Figure 1B), the mentor controlled the position and orientation (pose) of a stylus (Geomagic Touch; 3D Systems). The relative motion of the stylus was mapped to the 3D pointer rendered onto the live view of the operative field. Finally, the same stylus was also used to control the movements of the virtual tool (Figure 1C) based on our previous work [27].

**Figure 2.**
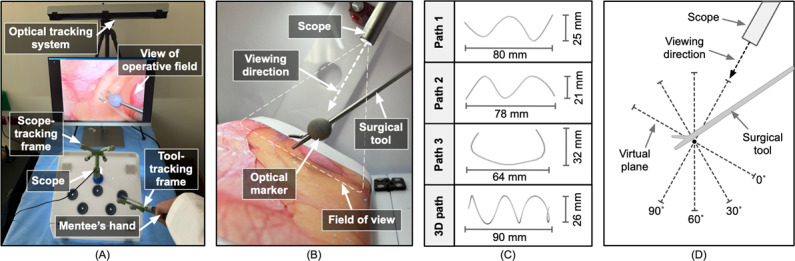
(A) Setup at mentee’s site comprising an optical tracking system, a screen rendering the view of the operative field, a box trainer, a surgical scope, and a laparoscopic instrument. (B) Setup inside the box trainer. (C) Three virtual 2D paths and one 3D path used in scenario A. (D) The orientation at which the 2D paths were rendered in front of the scope on a virtual plane.

At the mentee’s site, the study was performed on a low-fidelity synthetic phantom setup using a laparoscopic box trainer (Figure 2). The setup is shown in Figure 2A. A one-to-one comparison of the 3 dynamic AR cues requires a controlled environment that remains constant throughout the trials to eliminate confounding variables related to anatomical variability, tissue deformation, and procedural differences. For this reason, we designed this study using a phantom setup, which provides a stable and reproducible environment. This approach allowed for the isolation and objective assessment of the effect of each dynamic AR cue on tooltip movement and spatial guidance independent of the surgical procedure itself. Second, it avoided patient-related surgical risk and ethical concerns while still providing meaningful insights on the use of dynamic AR cues. The type of task that was selected was surgical tool maneuvering. It was chosen as it is an essential skill for laparoscopy requiring hand-eye coordination and precise instrument control [28]. Improvements in instrument control and spatial guidance are broadly transferable to more complex surgical tasks.

The dynamic AR visual cues were overlaid on the mentees’ view of the operative field in real time. The dynamic AR cues assisted the mentees in comprehending the required pose of the laparoscopic instrument and maneuvering it accordingly. To record the movements of the laparoscopic instrument with respect to the scope, tracking frames were used. An optical tracking system (V120-Trio; NaturalPoint) was used to track the motion of the laparoscopic instrument and the scope. In addition, the distal end of the laparoscopic instrument was fitted with an optical marker (Figure 2B). This assisted in capturing accurate positions of the laparoscopic instrument tip during the study. As the setup required placing an optical marker on the laparoscopic instrument, the user study was conducted in a simulated environment instead of on humans.

### Telementoring Scenarios

**Figure 3.**
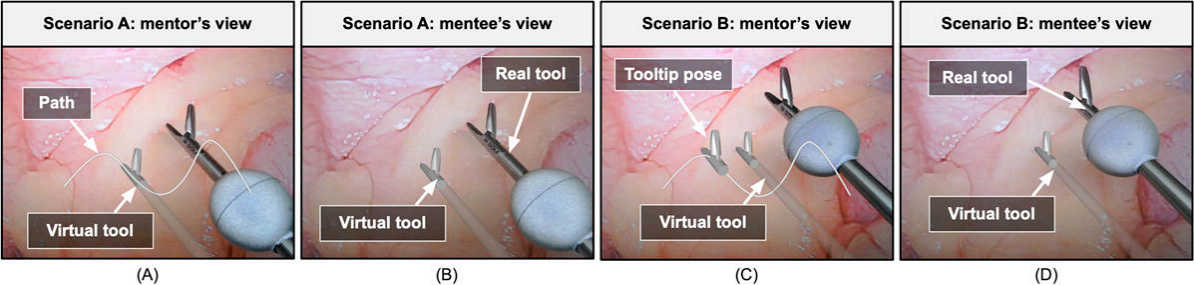
(A) Mentor’s view in scenario A. (B) Mentee’s view in scenario A. (C) Mentor’s view in scenario B. (D) Mentee’s view in scenario B.

Two scenarios, scenario A and scenario B, were simulated to assess the dynamic AR cues. Scenario A evaluated dynamic AR cues to facilitate continuous tool movements. The movements of the laparoscopic instrument were characterized by 4 different paths (Figure 2C). They consisted of 2D paths, each positioned at 0°, 30°, 60°, and 90° orientations (Figure 2D), and one 3D path. The paths were rendered onto the mentor’s screen (Figure 3A), whereas the mentees were able to see the dynamic AR cue generated by the mentor (Figure 3B). The mentor was asked to generate dynamic AR cues that would guide the mentees to follow the paths using the laparoscopic instrument. The mentees observed the dynamic AR cues in real time and performed the movements of the laparoscopic instrument to follow the mentor.

Scenario B evaluated dynamic AR cues to facilitate precise orientation of the laparoscopic instrument’s jaw. Six different feasible poses for orienting the jaw of the laparoscopic instrument were rendered to the mentor (Figure 3C). The mentor observed these poses and generated dynamic AR cues. The dynamic AR cues were rendered to the mentees (Figure 3D), and the mentees maneuvered the laparoscopic instrument to orient the jaw. As it was impossible to indicate this using 3D pointers, only hand gestures and virtual tools were used for guidance in scenario B.

In both scenarios, verbal instructions from the mentor were not provided. No verbal instructions were given to assess the dynamic AR cues independently as feedback in the form of audio cues can assist the mentees and alter the performance during the task. After completion of both scenarios, the mentees were required to complete the NASA Task Load Index (NASA-TLX) questionnaire [29] for each dynamic AR cue.

### Study Design and Outcomes Measured

We chose to study within-subject factors by having the same individuals participate in all 3 dynamic AR cues. In this way, the participants acted as their own controls, and we eliminated any variation in task performance arising from individual differences. Additionally, to eliminate bias arising from the learning effect, we randomized the order in which the AR cues were allocated to the participants, as well as the order of the different tasks performed using each AR cue. Before the start of the study, each mentee went through a preparatory step to familiarize themselves with the telementoring setup and dynamic AR cues. Each mentee performed both scenarios with all 3 dynamic AR cues. The sequence in which the dynamic AR cues were used by the mentor-mentee pair was randomized. Furthermore, in scenario A, the sequence in which the 2D and 3D paths were rendered to the mentor and the orientations in the case of the 2D path were also randomized. The primary outcomes measured as part of this study included objective assessments in the form of (1) the average dynamic time warping (DTW) distance between the paths followed by the mentees and the required paths shown by the mentor during scenario A and (2) angular errors (measured in degrees) in orienting the laparoscopic instrument jaw during scenario B. DTW is a technique for measuring similarity between time-series data using temporal alignment [30]. Unlike the Euclidean distance, which relies on point-to-point comparison, DTW allows for one-to-many and many-to-one comparison, which is useful for paths that vary in time and speed. DTW has been applied for surgical performance and instrument motion demonstrations [19,26,27,31]. A lower value of DTW indicates a greater similarity and better alignment between the mentees’ path and the reference path. The secondary outcome consisted of subjective assessment scores rated by the mentees using the NASA-TLX. These measured mental, physical, and temporal demands; effort; performance; and frustration on a scale from 1 to 10, with a lower score indicating a more positive rating.

### Statistical Analysis

In scenario A for paths 1 to 3, an ANOVA was performed to determine whether there were significant mean values over the 3 dynamic AR cues and the 4 orientations of the virtual plane. The dynamic AR cue model variable was highly statistically significant (*P*<.01) in all cases, whereas the orientation of the virtual plane was significant only in path 2. It was then decided to study all pairwise comparisons of the 3 dynamic AR cues for a path and a specified orientation, and as the same mentee participated in all 3 AR cues, we used the 2-tailed paired *t* test. For path 4, ANOVA established significant differences among the 3 AR cues. Therefore, a paired *t* test was used to examine statistically significant mean differences over all 3 pairs. Finally, in scenario B, a paired *t* test was used to determine the statistical significance between the 2 dynamic AR cues (hand gestures and virtual tool). In all the above models, the standard model assumptions were violated, and so the response variable was transformed using the natural logarithmic transformation to alleviate the problem. All relevant descriptive statistics and *P* values are reported in the Results section.

### Ethical Considerations

Institutional review board approval (MRC-03-23-786) was obtained from the Medical Research Center at Hamad Medical Corporation, Qatar. A research information sheet was used to obtain consent from the participants taking part in the study. All data collected was anonymized and stored securely. The participants enrolled in the study on a voluntary basis and were not provided any financial or other forms of compensation.

## Results

**Figure 4.**
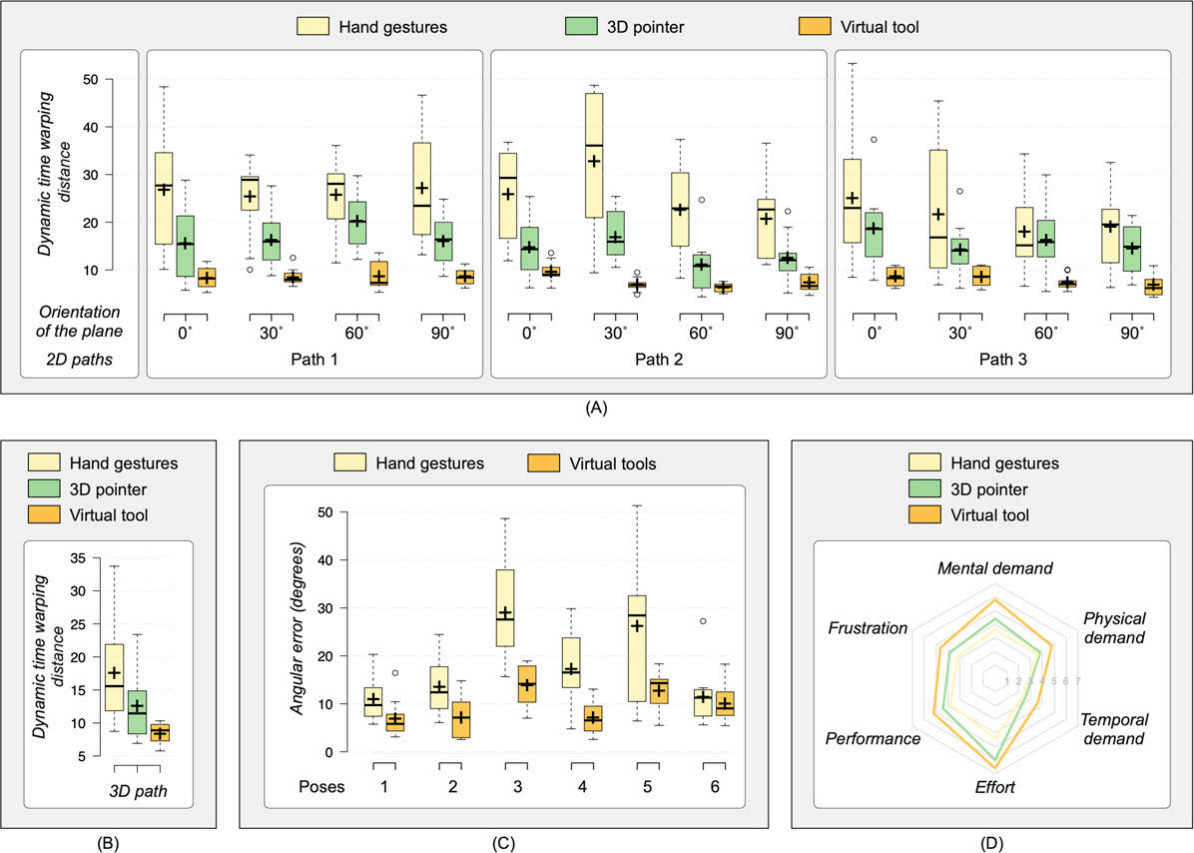
Average dynamic time warping distance from the intended path for each dynamic augmented reality cue while the mentees followed along: (A) three 2D paths at different orientations and (B) the 3D path during scenario A, (C) angular error orienting the jaw of the laparoscopic instrument for the 6 poses in scenario B, and (D) NASA Task Load Index scores reported by the mentees.

All participants successfully completed the tasks in both scenarios. Figure 4 presents the observed results when the mentees received guidance from the mentor in the form of dynamic AR cues. During scenario A, the paths followed by the mentees had improved DTW distances when they were guided by a virtual tool as opposed to hand gestures and a 3D pointer. As shown in Table 3, the reduction was statistically significant when the virtual tool was used by the mentor compared with hand gestures (*P*<.01 in 12/13, 92.3% of the trials) and the 3D pointer (*P*<.05 in 11/13, 84.6% of the trials). This improvement in performance was consistently observed across the three 2D paths in each plane orientation (Figure 4A), as well as the 3D path (Figure 4B).

**Table 3.** Dynamic time warping distance during scenario A and angular error (in degrees) during scenario B*.*

Scenario, path or pose, and virtual plane orientation	Hand gestures, mean (SD)	3D pointer, mean (SD)	Virtual tool, mean (SD)	*P* value		
				Hand gestures vs 3D pointer	3D pointer vs virtual tool	Virtual tool vs hand gestures
Scenario A—dynamic time warping distance						
Path 1						
0°	26.9 (11.9)	15.6 (7.3)	8.3 (2.4)	.048	.03	.001
30°	25.5 (8.2)	16.2 (5.6)	8.4 (1.8)	.03	.08	*<*.001
60°	25.8 (7.9)	20.2 (5.6)	8.6 (2.9)	.22	.001	*<*.001
90°	27.1 (12.0)	16.0 (5.3)	8.6 (1.6)	.03	.004	*<*.001
Path 2						
0°	25.9 (10.0)	14.8 (5.9)	9.6 (2.1)	.03	*<*.001	*<*.001
30°	32.8 (15.0)	16.9 (5.1)	6.9 (1.4)	.001	.06	.001
60°	22.6 (10.0)	11.1 (5.8)	6.4 (0.9)	.15	.03	*<*.003
90°	20.7 (8.0)	12.6 (5.0)	7.4 (2.1)	.03	.03	*<*.001
Path 3						
0°	25.1 (13.6)	18.8 (8.1)	8.7 (1.9)	.65	.004	.001
30°	21.7 (13.0)	14.3 (5.7)	8.6 (2.2)	.03	.001	*<*.001
60°	18.0 (8.6)	16.3 (7.0)	7.4 (1.5)	.04	.02	.001
90°	19.2 (8.8)	14.5 (5.4)	6.9 (2.3)	.17	.002	.03
3D path	17.6 (7.3)	12.6 (5.1)	8.4 (1.6)	.08	.03	*<*.001
Scenario B—angular error (degrees)						
Pose 1	11.0 (4.4)	—^a^	6.9 (4.0)	—	—	.03
Pose 2	13.5 (6.1)	—	7.2 (4.2)	—	—	.01
Pose 3	29.0 (10.4)	—	13.9 (4.3)	—	—	<.001
Pose 4	17.3 (8.1)	—	7.1 (3.4)	—	—	<.001
Pose 5	26.2 (15.0)	—	12.8 (4.5)	—	—	.03
Pose 6	11.5 (6.2)	—	10.1 (3.7)	—	—	.66

a Not applicable.

In scenario B, a substantial decrease in angular error was noted when the mentees were guided by the virtual tool as compared with hand gestures (*P*<.05 for 5 out of 6 poses). Pose 6, located toward the edge of the screen, did not have a statistically significant difference in angular error (Figure 4C).

The scores from the NASA-TLX (Figure 4D) suggest that the use of the 3D pointer and the virtual tool was relatively demanding for the mentees compared with hand gestures. However, the overall differences in NASA-TLX scores were not substantial (Table 4).

**Table 4.** Scores from the NASA Task Load Index questionnaire.

Item	Question	Scale	Hand gestures, mean (SD)	3D pointer, mean (SD)	Virtual tool, mean (SD)
Mental demand	“How mentally demanding were the tasks in both the scenarios?”	1‐10 (low-high)	3.6 (1.7)	4.4 (1.9)	5.8 (2.4)
Physical demand	“How physically demanding were the tasks in both the scenarios?”	1‐10 (low-high)	3.6 (2.3)	3.8 (2.0)	4.8 (2.8)
Temporal demand	“How hurried or rushed was the pace of the tasks in both the scenarios?”	1‐10 (low-high)	2.4 (1.5)	2.6 (1.3)	3.6 (1.5)
Performance	“How successful were you in accomplishing what you were asked to do?”	1‐10 (perfect-failure)	5.6 (2.3)	4 (1.2)	3.4 (2.1)
Effort	“How hard did you have to work to accomplish your level of performance?”	1‐10 (low-high)	3.8 (2.2)	4.4 (1.5)	5.2 (2.4)
Frustration	“How insecure, discouraged, irritated, stressed, and annoyed were you?”	1‐10 (low-high)	3.2 (1.8)	3.8 (1.3)	4.6 (1.8)

## Discussion

This study presents an assessment of 3 different dynamic AR cues, namely, hand gestures, a 3D pointer, and a virtual tool, used in telementoring during laparoscopic surgery. Integrating dynamic AR cues is necessary as static AR cues (telestrations) alone cannot fully reproduce the instructions conveyed during in-person guidance. The aim of this user study was to identify the most suitable dynamic AR cue for surgical telementoring. Overall, our results showed that the mentees were able to replicate the motions indicated by the mentor and follow the required path, demonstrating the feasibility of using dynamic AR cues. An important result of this study is that there was an improvement in performance when the mentor used the virtual tool to guide the mentees (as opposed to hand gestures or the 3D pointer). This was depicted in Figures 4A and B, where the nonoverlapping box plots across different plane orientations indicate a significant decrease in the DTW distance during scenario A. This indicates that the mentees could follow the required paths accurately in not only the 2D space but also the 3D space. As a result, it can be inferred that virtual tools would be useful during laparoscopic surgery. Such surgeries are performed in the insufflated body cavity with multiple tissue structures that vary in distance from the laparoscope’s distal end. Therefore, having cues that allow for depth perception has the potential to improve accuracy while reducing procedure time and incidence of errors [32]. Similarly, in scenario B, lower angular errors were noted for the poses guided by the virtual tool than for those guided by hand gestures. This is a valuable observation as the ability to communicate and reproduce the precise jaw orientation of a laparoscopic tool is essential for avoiding damage to vital anatomical structures during live surgery [4]. Examples of such surgical steps include placing the initial snip on tissue structure, cutting a vessel, or placing staples for incision closure. Virtual tools would be more efficient compared with hand gestures while guiding the mentee to easily communicate the optimal position and orientation of the sharp scissors used for cystic duct or artery dissection during cholecystectomy. The direction of the curved side of the sharp scissors with respect to anatomical landmarks can be conveyed effortlessly [4]. Similarly, the appropriate orientation of a harmonic scalpel (consisting of an active blade and inactive jaw) can also be conveyed while separating the gallbladder from the liver. Conveying such information is critical as incorrect handling can result in damage to organs and bleeding, compromising patient safety [33].

The proposed work is not intended to replace the use of static 2D cues such as annotations or telestrations during laparoscopic surgical telementoring. The optimal cue for a particular surgical telementoring session can vary with type and frequency of instructions that a mentor (expert surgeon) must convey to a mentee (operating surgeon). This is dependent upon the complexity of the surgical procedure as well as the difference in expertise level between the mentor and mentee. When the mentor and mentee are of comparable expertise, telementoring using audio or static AR cues would be sufficient. Dynamic AR cues can assist in gaining microskills particular to an unfamiliar surgical technique when the mentee already possesses sufficient macroskills such as anatomical expertise [19]. Additionally, a prior review of dynamic AR cues used in minimally invasive surgeries has noted extensive use of surgical tools for telementoring during suturing among medical students and the use of pointers during cholecystectomy procedures among surgical trainees [5]. Further studies with surgeons will help assess the impact of dynamic AR cues in various surgical scenarios in which significant differences in expertise exist between the mentor (expert surgeon) and mentee (operating surgeon).

Previous studies have shown that mentees report lower workload while receiving instructions using dynamic AR cues than using audio cues alone [3,7]. However, when assessing the workload perceived by the mentees for each of the dynamic AR cues in this study, there was no statistically significant difference between the NASA-TLX scores for hand gestures, the 3D pointer, or the virtual tool. Some of the mentees noted higher mental and physical demand while being guided by the virtual tool than by the other cues. This was attributed to the extra care taken by the mentee to align exactly with the mentor’s virtual tool. Although virtual tools might have resulted in a perceived higher demand, the results indicate that the mentees were able to understand the required position and orientation of the tool (which was not conveyed by the other cues). It is important to bear in mind that the use of the NASA-TLX as an outcome measure would receive a relatively lower rating according to the Medical Education Research Study Quality Instrument, indicating a weaker methodological quality, as it measures subjective user perceptions [34]. A reasonable approach would be to conduct multiple trials and observe the difference in perceived workload for each dynamic AR cue over the course of the trials.

Laparoscopic surgery is considered the standard approach for many procedures (such as cholecystectomy and colectomy) due to its advantage for the patient in terms of lower risk of complications and shorter recovery period [6]. Nevertheless, adoption of laparoscopic surgery continues to be limited worldwide due to its steep learning curve and the complex instrument-handling skills required. Given its clear advantage over the open approach, laparoscopic surgery might be initiated without in-person supervision in some countries [35,36]. However, nonmentored laparoscopic surgeries are associated with higher rates of conversion to open surgery and complications and longer hospital stays than mentored ones [36,37]. This is due to the challenges faced by inexperienced surgeons working with an operative view that is drastically different from that in open surgery, resulting in dissection of incorrect layers or improper placement of the initial incision [36]. Telementoring overcomes this challenge by connecting experienced surgeons with the operating surgeon. However, the amount of information that can be transferred between the experienced and operating surgeons is dependent upon the quality of the cues rendered. Dynamic AR cues enhance the information by depicting the exact tool-tissue interaction required during the surgery.

In this study, the dynamic AR cues used by the mentor were displayed to the mentees on a 2D screen. However, a typical surgical field consists of anatomical structures located at varying depth levels. With limited depth perception, it is challenging to accurately convey instructions, especially when the target anatomy is located deep within the operative field. For future work, it would be useful to incorporate multiple cameras to capture and render a 3D model of the mentor’s hand. In this way, the mentee will be able to perceive depth while being guided by hand gestures (which is lacking in the current setup). Furthermore, low-cost immersive head-mounted display (HMD) devices (such as the mixed reality–based Meta Quest 3 headset or Apple Vision Pro) could be used by the remote mentor to perceive a stereoscopic view of the surgical field obtained from a 3D laparoscopic camera used at the mentee’s side. This would also allow the expert surgeon to convey the dynamic AR cues in a 3D space and better demonstrate the required motions to the operating surgeon. In addition, instead of using a traditional bulky workstation, such as a laptop or desktop connected to external input devices to manipulate dynamic AR cues, these HMD devices incorporate built-in sensors that can directly track hand gestures. The captured hand movements can then be translated into dynamic AR cues in real time, eliminating the need for intermediary input devices. Furthermore, these HMD devices are portable and just need an internet connection to link to the operating room.

Several limitations and future directions should be considered when interpreting the findings of this study. First, this study was limited to 10 participants as mentees due to budget constraints. The primary aim of this study was exploratory in nature, and despite the small sample size, the findings provide preliminary evidence that may help inform and guide future large-scale studies. Second, while previously published articles have already established favorable results while using dynamic AR cues when compared with audio cues alone [6,9,23], it would be nevertheless useful to incorporate audio cues to simulate a real surgical training setting. The audio cues are useful when both mentor and mentee possess comparable surgical macroskills, such as anatomical knowledge, instrument maneuvering, and identification of critical structures. However, when a mentor remotely guides a less experienced mentee through a newly acquired surgical technique, the mentee may not yet have mastered technique-specific microskills, including visual tactility, economy of movement, and tissue handling. In such cases, the overlaid dynamic AR cues are expected to expedite skill acquisition by providing clear, unambiguous visual guidance independent of verbal instruction (audio cue). Third, implementing dynamic AR cues in a live surgical setting where objective clinical outcomes (such as operating time, error rate, or incidence of intraoperative complications) are measured will be required to fully evaluate the effectiveness of using the AR cues. While this study focused on laparoscopic surgery, it would be interesting to explore the application of AR cues for robot-assisted procedures where the surgical instruments have additional degrees of freedom.

In conclusion, this work has indicated that the use of virtual tools as dynamic AR cues results in improved performance compared with hand gestures and 3D pointers. However, there was no significant difference in the perceived workload among the 3 AR cues. Moving forward, further exploration on a multi-institutional scale is recommended. Studies integrating various dynamic AR cues during real-world surgery would also be essential to fully assess the AR cues’ clinical value during surgical telementoring.

## Abbreviations

AR: augmented reality
DTW: dynamic time warping
HMD: head-mounted display
NASA-TLX: NASA Task Load Index
